# Prevalence of Depressive Symptoms and Related Factors in Korean Employees: The Third Korean Working Conditions Survey (2011)

**DOI:** 10.3390/ijerph13040424

**Published:** 2016-04-14

**Authors:** Ji Nam Park, Mi Ah Han, Jong Park, So Yeon Ryu

**Affiliations:** 1 Department of Public Health, Graduate School of Health Science, Chosun University, 309 Pilmun-daero, Dong-gu, Gwangju 501-759, Korea; pjn1027@naver.com; 2 Department of Preventive Medicine, College of Medicine, Chosun University, 309 Pilmun-daero, Dong-gu, Gwangju 501-759, Korea; jpark@chosun.ac.kr (J.P.); canrsy@chosun.ac.kr (S.Y.R.)

**Keywords:** related factors, employee, working conditions survey, depression

## Abstract

The aim of this study was to analyze the association between general working conditions and depressive symptoms among Korean employees. The target population of the study was native employees nationwide who were at least 15 years old, and 50,032 such individuals were enrolled in the study. Depressive symptoms was assessed using the WHO-5 wellbeing index. Associations between general characteristics, job-related characteristics, work environment, and depressive symptoms were tested using chi-square tests, t-tests, and multiple logistic regression analysis. The prevalence of depressive symptoms was 39% (40.7% in males and 36.5% in females). Multiple regression analysis revealed that male subjects, older subjects, subjects with higher education status, subjects with lower monthly income, current smokers, and frequent drinkers were more likely to have depressive symptoms. In addition, longer weekly work hours, occupation type (skilled, unskilled, operative, or economic sector), shift work, working to tight deadlines, exposure to stress at work, and hazard exposure were associated with depressive symptoms. This representative study will be a guide to help manage depression among Korean employees. We expect that further research will identify additional causal relationships between general or specific working conditions and depression.

## 1. Introduction

Depression is a debilitating condition that places a huge health burden on society. The World Health Organization (WHO) ranks it as the leading cause of disability worldwide [1]. The most recent Korean epidemiological survey of mental disorders estimates that the lifetime prevalence of depression is 6.7% [2]. Depression is a major health issue among Korean employees [3,4]. Many aspects of life, especially in the workplace, influence depression, which may in turn weaken productivity and overall job performance. Data from the fourth European Working Conditions Survey show that employees who spend more time at work have a higher prevalence of depression than those who spend less time at work. Employees who experienced depression in the previous year are more likely to work a full week [5]. Many studies of the link between depression and shift work have found that the prevalence of depression is higher among employees who often work at night compared with those working regular schedules [6].

Furthermore, occupational conditions such as job strain, demand, and stress are also associated with depression. High strain can be described as high work demands that exceed an employee’s abilities, and this increases stress and risk factors that influence depression [7]. Several cross-sectional studies have found a relationship between negative occupational conditions and poor mental health outcomes [8,9,10].

Several previous studies evaluated depression and related factors in Korean employees. One such study reports that 7.4% of the subjects responded that they had been diagnosed as having depression from medical professionals. The conclusion was that this lower prevalence might be due to a lower awareness or diagnosis rate compared to other countries rather than an actual lower rate of depression. These results suggest that depression is poorly recognized and not disclosed to employers in the Korean workplace [11]. Most of these studies are, however, targeted at populations from a certain city or province using convenience sampling. The aim of this study was, therefore, to analyze the prevalence of depressive symptoms and related factors, and their association, among Korean employees using the third Korean Working Conditions Survey (KWCS).

## 2. Methods

### 2.1. Data Sources

This study used data collected from the third KWCS, which was conducted by the Occupational Safety and Health Research Institute (OSHRI) and the data are freely available upon request. The target population of the KWCS was those who were employed and aged ≥15 years, and was selected from across the nation using multistage systematic cluster sampling methods. The KWCS was conducted through face-to-face interviews during house-to-house visits. If there were more than two qualified employees in each household, trained interviewers interviewed only people who were closest to the date of birth from the research date. The KWCS collected data about each employee’s general characteristics, work-related characteristics, work environment, and health status. All subjects gave their informed consent for inclusion before they participated in the study. The final sample size in the third KWCS was 50,032 native employees [12]. The cooperation rate of 66.2%, contact rate of 56.6%, refusal rate of 18.0% and response rate of 35.4% were calculated by the definition of the American Association for Public Opinion Research [13].

### 2.2. Variables

#### 2.2.1. General Characteristics

The general characteristics were gender, age (15–29, 30–39, 40–49, or ≥50 years), education level (≤middle school, high school, or ≥college), monthly income (<150, 151–299, or ≥300 × 10,000 won), smoking status (never, former, or current), drinking frequency (non-drinker, 1–4 times/month, or ≥2 times/week), and self-rated health (good, or fair/poor). 

#### 2.2.2. Work-Related Characteristics

Work-related characteristics consisted of weekly working hours (<40, 40–48, or ≥49 h), type of occupation, duration of career (<1, 1–5, or ≥6 years), shift work (yes or no), and work environment. The type of occupation was classified into the following four categories according to the Korean standard classification of occupations. The first occupational group includes managerial, professional, and clerical occupations. The second group includes service and sales occupations. The third group includes skilled, unskilled, and operative occupations. The fourth group included types of economic sectors, such as agriculture, fishing, and mining; manufacturing; construction; service; and others [14].

The characteristics of work environments that were included were working at very high speed, working to tight deadlines, exposure to stress at work, and hazard exposure. Hazard exposure was assessed as the sum of an employee’s responses to nine questions and classified for physical factors such as vibration, noise, high and low temperature, breathing in smoke, fumes, powder, vapors, chemical products, or tobacco smoke from other people, and infectious substances [14]. The possible answers to these questions and the corresponding number of points were: ‘’all of the time’’ (7), ’’almost all of the time’’ (6), ‘’around three-quarters of the time’’ (5), ‘’half of the time’’ (4), ‘’around a quarter of the time’’ (3), ‘’almost never’’ (2), and ‘’never’’ (1). A seven-level Likert scale was used in which high scores denote high risk.

#### 2.2.3. Depressive Symptoms

Depressive symptoms was assessed using the WHO-5 Wellbeing Index. The WHO-5 Wellbeing Index shows good internal and external validity. The WHO-5 instrument is well known and is useful in the context of depression research [15,16]. Employees were asked to rate five statements depending on how close they were to how they had been feeling over the last two weeks. The statements were: “I have felt cheerful and in good spirits”, “I have felt calm and relaxed”, “I have felt active and vigorous”, “I woke up feeling fresh and rested”, and “My daily life has been filled with things that interest me“. The raw score was computed by totaling the score of the response to each statement. The possible answers to these statements and the corresponding number of points were: “all of the time” (5), “most of the time” (4), “more than half of the time” (3), “less than half of the time” (2), “some of the time” (1), and “at no time” (0). A raw score up to and including 12 represents a high-risk status for depression, and a score of 13 and above represents a normal status.

#### 2.2.4. Data Analysis

All data analyses were performed using SPSS software (version 23, SPSS Inc., Chigaco, IL, USA). Simple descriptive statistical analyses were used to obtain frequencies and percentages for general and work-related characteristics. Work environment scores were reported as mean and standard deviation. Depressive symptoms-related overall characteristics were analyzed using chi-square tests and *t*-tests. Finally, multiple logistic regression analysis was used to identify depressive symptoms-related factors. Additionally, a stratified analysis by sex was performed to examine the consistency of related factors with depressive symptoms.

## 3. Results

### 3.1. Depressive Symptoms by Subjects’ General Characteristics

The overall average score for depression was 13.7 ± 5.30. According to the categorization of risk status, 39% of the total population had depressive symptoms. The prevalence of depression in men (40.7%) was significantly higher than that in women (36.5%, *p* < 0.001). When age group was considered, the prevalence of depression among subjects ≥50 years and 15–29 years was 46.0% and 31.3%, respectively (*p* < 0.001). Also, education level, monthly income, smoking status, drinking frequency, and self-rated health were significantly associated with depressive symptoms (Table 1).

### 3.2. Depressive Symptoms by Work-Related Characteristics

The prevalence of depressive symptoms among those with ≥49 weekly work hours (41.3%) was statistically higher than among those who worked on average 40–48 h per week (35.7%, *p* < 0.001). With regard to type of occupation, a higher proportion of people who work in the economic sector were depressed (51.0%) compared with people working in managerial, professional, and clerical professions (30.7%, *p* < 0.001). Individuals with a career duration of ≥6 years (40.9%) had a significantly higher prevalence of depressive symptoms than those with careers of a shorter duration. A statistically significant proportion of the population performing shift work exhibited depressive symptoms (43.6%, *p* < 0.001), compared with those that did not perform shift work. Regarding work environment, the average score for “working to tight deadlines” was 2.50 ± 1.54 in the group of depressed individuals, and 2.57 ± 1.72 in the normal group. The average score for exposure to stress at work in the group with depressive symptoms was 3.00 ± 0.94, and for the normal group the average score was 2.89 ± 1.01. For hazard exposure, the average score for those with depressive symptoms was 16.02 ± 7.10, and for the normal group the average was 14.32 ± 6.82 (Table 2).

### 3.3. Multiple Logistic Regression Analysis of Risk Factors for Depressive Symptoms

Multiple logistic regression of risk factors related to depressive symptoms revealed that males had greater odds than females (OR = 1.09, 95% CI: 1.03–1.15). The odds for employees who were over 50 years old were higher than for those who were 15–29 years old (OR = 1.14, 95% CI: 1.06–1.23). Middle school-level education was associated with higher odds for depressive symptoms compared with college-level education (OR = 1.54, 95% CI: 1.43–1.67). Individuals with a monthly income of <150 was associated with higher odds for depressive symptoms than those with a monthly income of >300 (OR = 1.19, 95% CI: 1.11–1.28). Current smokers were more likely to have depressive symptoms than non-smokers (OR = 1.18, 95% CI: 1.11–1.24). Employees who drank more than two times a week had greater odds for depressive symptoms than non-drinkers (OR = 1.09 95% CI: 1.02–1.15). People with skilled, unskilled, or operative jobs had higher odds for depressive symptoms than those with managerial, professional, and clerical jobs (OR = 1.21, 95% CI: 1.13–1.29). The odds ratio for employees performing shift work was 1.12, 95% CI: 1.04–1.20. Psychosocial factors such as “working to tight deadlines” (OR = 0.94, 95% CI: 0.93–0.95), being “exposed to stress in your work” (OR = 1.16, 95% CI: 1.13–1.18), and hazard exposure (OR = 1.02, 95% CI: 1.01–1.02) were associated with depressive symptoms (Table 3).

In the stratified analysis by sex, education level, smoking status, drinking frequency, self-rated health, working to tight deadlines, exposure to stress at work, and hazard exposure were factors associated with depressive symptoms in both sexes (Table 4).

## 4. Discussion

Depression is a significant public health concern worldwide. Recognizing its causes can help reduce its impact on individual lives and society. This study analyzed the prevalence of depressive symptoms and related factors among Korean employees.

This representative study found the prevalence of depressive symptoms was 39% among employed workers. In a previous study (the fifth European Working Conditions Survey, 2011), more than 23% of workers in Europe reported low levels of wellbeing, indicating that they should be assessed for depression [17]. In the second KWCS (2006), the prevalence of depressive symptoms among Korean employees was 40.3% [18]. The prevalence of depressive symptoms among employees is higher than in any other population group. Most studies using the WHO-5 as a psychiatric measuring tool report higher depression prevalence rates than studies using other psychiatric measurements. The WHO-5 has good internal and external validity, although it is a short screening instrument used for the detection of depression [15,19].

In a European Working Conditions Survey (2010), mean weekly working hours were 40.87 ± 8.3 for men and 36.89 ± 9.1 for women [20] and the proportion of long working hours (defined as ≥48 h per week) was 20.3% in men and 10.0% in women [17]. On the other hand, in this study weekly working hours were 49.5 ± 15.1 and the proportion of workers who worked more than 49 weekly work hours was 45.3%. The employees with good self-rated health were 77.8% of men and 74.0% of women in the European Working Conditions Survey [20]. In this study, 69.0% of employees had good self-rated health. So, Korean employees work relatively longer hours and have poorer self-rated health.

The current representative study revealed differences between the genders in terms of depressive symptoms. In a previous study, women were more prone to suffer from depression than men [21]. However, the proportion of depressive symptoms in men was significantly higher than that in women in this study. Male workers are more willing to work in dangerous environments [22]. All in all, men were exposed to more stress at work, longer working hours, and more hazardous working conditions than women in this study (data not shown). Older employees are more likely to have depressive symptoms. This is because they are more likely to have health problems than their younger counterparts so chronically stressful conditions may have a more negative effect on their health. Lower levels of education and monthly income were associated with a higher risk of depressive symptoms. In terms of monthly income and education, inequalities in socioeconomic status also influence the prevalence of depression [23].

This study also showed that current smokers and heavy drinkers have a higher prevalence of depressive symptoms than non-smokers and non-drinkers. These results are consistent with previous findings. The main adverse effect of nicotine is addiction, which has a negative effect on depression. This may be because nicotine binds to certain receptor molecules on the surface of nerve cells in the brain, and these interactions increase depressive symptoms. Alcohol is associated with a depressant effect, meaning that it disrupts the biological balance. Alcohol may affect our thoughts, feelings, and actions. Consequently, long-term mental health is worsened so that depression co-occurs with other serious medical illnesses. On the other hand, employees who are stressed and depressed use smoking or drinking to relieve feelings of stress [24,25].

Working long hours may increase job-specific strain, conflict, and neglect of important relationships at outside of work [26]. Working long hours, therefore, significantly increases the risk of depression. An employee’s type of occupation is associated with depressive symptoms. People who work in non-professional and unskilled jobs tend to have more stress than those in professional jobs. A previous study found that non-professional and routine jobs have higher physical demands and require more time spent working. Work-related stress gradually increases as the physical reaction to those demands take place [27]. A previous study also indicates that shift workers are more vulnerable to stress than non-shift workers. This is because shift work increases the risk for sleep disorders, thereby producing worse health consequences [28].

The effect of stress on depression is well known. Stress often lowers mood, which can lead to impaired concentration so that direct or indirect effects of stress can turn into depression. Stress also has a significant effect on work productivity and performance [29]. Many recent studies have focused on hazardous factors. Exposure to hazardous physical factors causes harmful effects on health. In addition, hazardous exposures may lead to accidents and cumulatively induce stress [14]. Consequently, physically stressful conditions produce psychological distress. So, reducing exposure of stress or hazardous factors will be helpful to prevent depressive symptoms.

In the stratified analysis by sex, the associations were similar to the results with the total sample. However, the associations of several factors with depressive symptoms differed from the results of the analysis with the total sample. Factors associated with depressive symptoms differed between men and women and these results suggested that different gender-specific strategies are needed to control depression.

This study had several limitations. Not only is depression associated with job-related characteristics and work environment, but it also has a relationship with social and supervisory factors. This study was, however, unable to examine such factors due to a lack of data. Because this study is based on a cross-sectional study, it was difficult to examine cause and effect relationships. So, working long hours can affect the development of depressive symptoms in employees. On the other hand, depressive symptoms influenced the likelihood of employees working long hours. Further prospective study is helpful to clarify the time–event relationship. 

## 5. Conclusions 

This study is a good example for assessing overall depressive symptoms and related factors among Korean employees, because the target population was employees nationwide. Furthermore, this study will help improve work-related environments and set depression management guidelines for Korean employees.

## Figures and Tables

**Table 1:** Association between depressive symptoms and general characteristics.

Characteristic	Classification	Total	Depressive Symptoms		*p*-Value
			Yes	No	
Total		*N* = 50,032	19,530 (39.0)	30,502 (61.0)	
Gender	Male	29,138 (58.2)	11,887 (40.7)	17,252 (59.3)	<0.001
	Female	20,894 (41.8)	7643 (36.5)	13,251 (63.5)	
Age (years)	15–29	7842 (15.7)	2455 (31.3)	5387 (68.7)	<0.001
	30–39	11,784 (23.6)	4052 (34.4)	7732 (65.6)	
	40–49	13,641 (27.3)	5314 (39.0)	8327 (61.0)	
	≥50	16,765 (33.5)	7708 (46.0)	9056 (54.0)	
Education	≤Middle school	8201 (16.4)	4265 (52.0)	3937 (48.0)	<0.001
	High school	19,284 (38.5)	8145 (42.2)	11,139 (57.8)	
	≥College	22,547 (45.1)	7120 (31.6)	15,427 (68.4)	
Monthly income (10,000 won)	<150	16,384 (32.7)	7076 (43.2)	9308 (56.8)	<0.001
	151–299	22,641 (45.3)	8657 (38.2)	13,984 (61.8)	
	≥300	10,987 (22.0)	3792 (34.5)	7196 (65.5)	
Smoking status	Never	27,616 (55.2)	10,088 (36.5)	17,528 (63.5)	<0.001
	Former	5805 (11.6)	2348 (40.4)	3457 (59.6)	
	Current	16,611 (33.2)	7094 (42.7)	9518 (57.3)	
Drinking frequency	Non-drinker	12,813 (25.6)	5171 (40.4)	7643 (59.6)	<0.001
	1–4 times/month	24,082 (48.1)	8632 (35.8)	15,450 (64.2)	
	≥2 times/week	13,137 (26.3)	5727 (43.6)	7410 (56.4)	
Self-rated health	Good	34,537 (69.0)	10,981 (31.8)	23,555 (68.2)	<0.001
	Fair/poor	15,495 (31.0)	8548 (55.2)	6947 (44.8)	

Data are expressed as number (%).

**Table 2:** Association between depressive symptoms and work-related characteristics.

Characteristics	Classification	Total	Depressive Symptoms		*p*-Value
			Yes	No	
Weekly work hours	<40	5645 (11.3)	2409 (42.7)	3236 (57.3)	<0.001
	40–48	21,702 (43.4)	7743 (35.7)	13,959 (64.3)	
	≥49	22,685(45.3)	9378 (41.3)	13,307 (58.7)	
Type of occupation	Managerial, professional, clerical	14,360 (28.6)	4408 (30.7)	9951 (69.3)	<0.001
	Service & sales	17,433 (34.8)	6464 (37.1)	10,965 (62.9)	
	Skilled, unskilled, operative	14,800 (29.7)	6901 (46.6)	7902 (53.4)	
	Economic sector	3439 (6.9)	1756 (51.0)	1684 (49.0)	
Duration of career (years)	<1	6182 (12.4)	2321 (37.5)	3861 (62.5)	<0.001
	1–5	22,324 (44.6)	8409 (37.7)	13,915 (62.3)	
	≥6	21,526 (43.0)	8800 (40.9)	12,726 (59.1)	
Shift work	No	46,391 (92.7)	17,943 (38.7)	28,447 (61.3)	<0.001
	Yes	3641 (7.3)	1586 (43.6)	2055 (56.4)	
Work environment					
Working at very high speed		2.75 ± 1.69	2.74 ± 1.58	2.76 ± 1.76	0.161
Working to tight deadlines		2.54 ± 1.65	2.50 ± 1.54	2.57 ± 1.72	<0.001
Exposure to stress at work		2.93 ± 0.99	3.00 ± 0.94	2.89 ± 1.01	<0.001
Hazard exposure		14.98 ± 6.98	16.02 ± 7.10	14.32 ± 6.82	<0.001

Data are expressed as number (%) or mean ± standard deviation.

**Table 3:** Odds ratios (95% CI) for depressive symptoms.

Characteristic	OR (95% CI)
Gender (/female)	
Male	1.09 (1.03–1.15)
Age (/15–29)	
30–39	1.07 (1.01–1.14)
40–49	1.18 (1.10–1.26)
≥50	1.14 (1.06–1.23)
Education (/≥college)	
≤Middle school	1.54 (1.43–1.67)
High school	1.28 (1.22–1.35)
Monthly income (/>300)	
<150	1.19 (1.11–1.28)
151–299	1.11 (1.06–1.18)
Smoking status (/never)	
Former	1.06 (0.99–1.13)
Current	1.18 (1.11–1.24)
Drink frequency (/none)	
1–4 times/month	0.91 (0.87–0.96)
≥2 times/week	1.09 (1.02–1.15)
Self-rated health (/good)	
Fair/poor	2.35 (2.26–2.45)
Weekly work hours (/40–48)	
<40	1.01 (0.95–1.09)
≥49	0.99 (0.95–1.03)
Type of occupation (/managerial, professional, clerical)	
Service & sales	1.04 (0.98–1.10)
Skilled, unskilled, operative	1.21 (1.13–1.29)
Economic sector	1.05 (0.96–1.16)
Duration of career (/<1)	
1–5	1.07 (1.01–1.14)
≥6	0.98 (0.92–1.05)
Shift work (/no)	
Yes	1.12 (1.04–1.20)
Working to tight deadlines	0.94 (0.93–0.95)
Exposure to stress at work	1.16 (1.13–1.18)
Hazard exposure	1.02 (1.01–1.02)

**Table 4:** Odds ratios (95% CI) for depressive symptoms according to sex.

Characteristic	Male	Female
Age (/15–29)		
30–39	1.26 (1.15–1.38)	0.94 (0.85–1.04)
40–49	1.45 (1.32–1.60)	0.98 (0.89–1.08)
≥50	1.35 (1.22–1.48)	1.01 (0.90–1.13)
Education (/≥college)		
≤Middle school	1.43 (1.30–1.58)	1.73 (1.53–1.95)
High school	1.26 (1.18–1.34)	1.36 (1.25–1.47)
Monthly income (/>300)		
<150	1.27 (1.16–1.39)	1.10 (0.98–1.23)
151–299	1.14 (1.07–1.21)	1.05 (0.94–1.17)
Smoking status (/never)		
Former	1.12 (1.04–1.21)	0.83 (0.69–0.99)
Current	1.24 (1.17–1.32)	0.90 (0.79–1.03)
Drink frequency (/none)		
1–4 times/month	0.94 (0.88–1.02)	0.89 (0.84–0.95)
≥2 times/week	1.09 (1.01–1.17)	1.17 (1.06–1.30)
Self-rated health (/good)		
Fair/poor	2.37 (2.24–2.50)	2.34 (2.19–2.49)
Weekly work hours (/40–48)		
<40	1.09 (0.98–1.21)	0.95 (0.87–1.04)
≥49	1.02 (0.97–1.08)	0.93 (0.86–0.99)
Type of occupation (/managerial, professional, clerical)		
Service & sales	1.11 (1.03–1.20)	0.97 (0.89–1.06)
Skilled, unskilled, operative	1.28 (1.18–1.38)	1.08 (0.97–1.21)
Economic sector	1.05 (0.92–1.19)	1.07 (0.91–1.24)
Duration of career (/<1)		
1–5	0.99 (0.90–1.08)	1.14 (1.05–1.25)
≥6	0.91 (0.83–1.01)	1.02 (0.92–1.13)
Shift work (/no)		
Yes	1.09 (1.00–1.19)	1.16 (1.02–1.33)
Working to tight deadlines	0.95 (0.94–0.97)	0.92 (0.91–0.94)
Exposure to stress at work	1.17 (1.14–1.20)	1.14 (1.10–1.17)
Hazard exposure	1.02 (1.02–1.03)	1.02 (1.02–1.03)

## References

[1] Smith K. (2014). Mental health: A world of depression. Nature.

[2] Jeon H.J. (2012). Epidemiologic studies on depression and suicide. J. Korean Med. Assoc..

[3] Cho J.J., Kim J.Y., Chang S.J., Fiedler N., Koh S.B., Crabtree B.F., Kang D.M., Kim Y.K., Choi Y.H. (2008). Occupational stress and depression in Korean employees. Int. Arch. Occup. Environ. Healt..

[4] Choi K.S., Kang S.K. (2010). Occupational psychiatric disorders in Korea. J. Korean Med. Sci..

[5] Pascal P., Damien M. (2001). Third European Survey on Working Conditions 2000.

[6] Adams P.F., Kirzinger W.K., Martinez M.E. (2012). Summary Health Statistics for the U.S. Population: National Health Interview Survey, 2011.

[7] Mausner-Dorsch H., Eaton W.W. (2000). Psychosocial work environment and depression: Epidemiologic assessment of the demand-control model. Am. J. Public Health.

[8] Godin I., Kittel F. (2004). Differential economic stability and psychosocial stress at work: Associations with psychosomatic complaints and absenteeism. Soc. Sci. Med..

[9] Sanne B., Mykletun A., Dahl A.A., Moen B.E., Tell G.S. (2005). Testing the Job Demand-Control-Support model with anxiety and depression as outcomes: The Hordaland Health Study. Occup. Med..

[10] Laaksonen M., Rahkonen O., Martikainen P., Lahelma E. (2006). Associations of psychosocial working conditions with self-rated general health and mental health among municipal employees. Int. Arch. Occup. Environ. Health.

[11] Hong J.P., Lee D., Sim Y., Kim Y.H. (2015). Awareness, attitude and impact of perceived depression in the workplace in Korea. J. Korean Neuropsychiatr. Assoc..

[12] Yoo M., Lee S., Kang M.Y. (2015). Gender and educational level modify the relationship between workplace mistreatment and health problems: A comparison between South Korea and EU countries. J. Occup. Health.

[13] Kim H.C., Lamichhane D.K., Jung D.Y., Kim H.R., Choi E.H., Oh S.S., Kang H.T., Rhee K.Y., Chang S.J. (2015). Association of active and passive smoking with occupational injury in manual workers: A cross-sectional study of the 2011 Korean working conditions survey. Ind. Health.

[14] Rhee K.Y., Kim Y.S., Cho Y.H. (2015). The type of payment and working conditions. Saf. Health Work.

[15] Bonsignore M., Barkow K., Jessen F., Heun R. (2001). Validity of the five-item WHO Well-Being Index (WHO-5) in an elderly population. Eur. Arch. Psychiat. Clin. Neurosci..

[16] Krieger T., Zimmermann J., Huffziger S., Ubl B., Diener C., Kuehner C., Grosse Holtforth M. (2014). Measuring depression with a well-being index: Further evidence for the validity of the WHO Well-Being Index (WHO-5) as a measure of the severity of depression. J. Affect. Disord..

[17] Schutte S., Chastang J.F., Malard L., Parent-Thirion A., Vermeylen G., Niedhammer I. (2014). Psychosocial working conditions and psychological well-being among employees in 34 European countries. Int. Arch. Occup. Environ. Health.

[18] Lee B.J., Park S.G., Min K.B., Min J.Y., Hwang S.H., Leem J.H., Kim H.C., Jeon S.H., Heo Y.S., Moon S.H. (2014). The relationship between working condition factors and well-being. Ann. Occup. Environ. Med..

[19] Topp C.W., Ostergaard S.D., Sondergaard S., Bech P. (2015). The WHO-5 Well-Being Index: A systematic review of the literature. Psychother. Psychosom..

[20] Lunau T., Bambra C., Eikemo T.A., van der Wel K.A., Dragano N. (2014). A balancing act? Work-life balance, health and well-being in European welfare states. Eur. J. Public Health.

[21] Schmaus B.J., Laubmeier K.K., Boquiren V.M., Herzer M., Zakowski S.G. (2008). Gender and stress: Differential psychophysiological reactivity to stress reexposure in the laboratory. Int. J. Psychophysiol..

[22] Rommel A., Varnaccia G., Lahmann N., Kottner J., Kroll L.E. (2016). Occupational Injuries in Germany: population-wide national survey data emphasize the importance of work-related factors. PLoS ONE.

[23] Lorant V., Deliege D., Eaton W., Robert A., Philippot P., Ansseau M. (2003). Socioeconomic inequalities in depression: A meta-analysis. Am. J. Epidemiol..

[24] Kouvonen A., Kivimaki M., Virtanen M., Pentti J., Vahtera J. (2005). Work stress, smoking status, and smoking intensity: An observational study of 46,190 employees. J. Epidemiol. Community Health.

[25] Azagba S., Sharaf M.F. (2011). The effect of job stress on smoking and alcohol consumption. Health Econ. Rev..

[26] Milner A., Smith P., LaMontagne A.D. (2015). Working hours and mental health in Australia: Evidence from an Australian population-based cohort, 2001–2012. Occup. Environ. Med..

[27] Daraiseh N., Genaidy A.M., Karwowski W., Davis L.S., Stambough J., Huston R.I. (2003). Musculoskeletal outcomes in multiple body regions and work effects among nurses: The effects of stressful and stimulating working conditions. Ergonomics.

[28] Schwartz J.R., Roth T. (2006). Shift work sleep disorder: Burden of illness and approaches to management. Drugs.

[29] Woo J.M., Kim W., Hwang T.Y., Frick K.D., Choi B.H., Seo Y.J., Kang E.H., Kim S.J., Ham B.J., Lee J.S. (2011). Impact of depression on work productivity and its improvement after outpatient treatment with antidepressants. Value Health.

